# Integrated Optomechanical Analysis of the Impact of an Ø 800 mm Primary Mirror on the Imaging Quality of an Optical System

**DOI:** 10.3390/s25185759

**Published:** 2025-09-16

**Authors:** Ruijing Liu, Yi Zhang, Yu Liu, Qingya Li

**Affiliations:** 1 Department of Intelligent Manufacturing Engineering, School of Mechanical and Vehicle Engineering, Changchun University, Changchun 130022, China; liuruij0326@126.com (R.L.); zhangyi3612024@126.com (Y.Z.); a1223912419@163.com (Y.L.); 2 Changchun Institute of Optics, Fine Mechanics and Physics (CIOMP), Chinese Academy of Sciences, Changchun 130033, China

**Keywords:** space optical telescope, primary mirror, modulation transfer function (MTF), optical–mechanical analysis

## Abstract

With the rapid advancements that are occurring in space technology, there is an increasing demand for improvements in the image quality of high-resolution space optical telescopes. The optical performance of the primary mirror plays a crucial role in determining the overall image quality of these optical systems. In this study, we analyze the rigid-body displacement and mirror deformation of an optical mirror in terms of the entire satellite hierarchy, utilizing integrated optomechanical analysis methods to assess the modulation transfer function (MTF) of the optical system. Additionally, we simulate MTF degradation under gravitational effects. Further, we conduct an experimental optical detection test on the main mirror assembly to validate our simulation analysis. This study provides valuable insights into structuring whole-satellite layouts and designing mirror support structures.

## 1. Introduction

Space optical remote sensing technology is extensively utilized in various fields, including resource exploration and terrain surveying. In response to the pressing demand for high-quality remote sensing images, high-resolution spatial optical telescopes have been developed. In a high-resolution optical system, the large-aperture primary mirror serves as a critical component that must endure complex and stringent static and dynamic challenges throughout processing, assembly, transportation, launch, and in-orbit operations [1]. Under these intricate conditions, the structural response of the mirror can lead to issues such as tilt, decenter, and deformation. These problems may result in optical aberrations and jitter errors that adversely affect both imaging quality and positioning accuracy within the optical system; in severe cases, they could even prevent successful imaging altogether [2]. As a key element of an optical telescope, the primary mirror must maintain excellent static and dynamic performance to ensure that high-quality images can be produced.

Many scholars have employed the method of optical machine integration to analyze the performance of optical systems. Myung Cho et al. conducted an analysis of the optomechanical structural deformations of a particular telescope under both static and dynamic loads [3]. They employed the sensitivity equation of the line-of-sight (LOS) error to evaluate the influence of optomechanical structural deformations on the optical system. Shi Jianliang et al. developed an optomechanical interface program: this program was founded on the Zernike polynomial fitting algorithm and capable of facilitating data interaction [4]. They further analyzed the impact of micro-vibrations on the optical performance of a certain telescope. Furthermore, Liu Guang established a data interface between the optical model and the structural model using the Zernike polynomial fitting method [5]. This interface allowed for the importation of rigid-body displacements and surface form errors of the optical mirror under the influence of external environments into optical analysis software. Liu Guang also investigated the effects of the key structural and thermal parameters of a space optical camera on the imaging quality of its optical system, providing guidance for the structural design of camera components. Xu Guangzhou et al. developed the optomechanical interface program ISIA based on the Zernike polynomial fitting algorithm and the Gram–Schmidt algorithm [6]. Their aim was to analyze the influence of external loads on the optical performance of a space solar telescope. Leveraging the results of optomechanical simulations, they offered data-based references for the structural design of the space solar telescope, thereby enhancing its environmental adaptability.

Joao Faria et al. underscored the significance of using the integrated analysis method in the case of optical machines, examining how temperature variations affect the imaging quality of an optical telescope’s imaging system by integrating Ansys and Zemax with Sigfit [7]. To minimize wavefront error (WFE) in control systems, numerous international researchers conduct integrated optomechanical analysis using Sigfit, utilizing optimized analytical results to inform detailed designs of optic-machine systems [8,9]. Wenqiang Yang developed a MATLAB-based interface program for integrated optic-machine simulation data [10]. This program determined Zernike fitting coefficients and imported fitted data into Zemax for image quality assessment. Mengqi Shao successfully conducted an integrated analysis of optical and mechanical systems by utilizing Isight to create an interface with NX10.0 , OptiStruct (2017), and various other analytical software tools [11]. This approach optimized both the structure and dimensions of spatial optical telescopes to reduce line-of-sight axis displacement and wavefront errors while ensuring high imaging quality. Zhidong Hu et al. utilized Workbench, Sigfit, and Zemax to perform an integrated analysis of a specific optical machine configuration [12]. They investigated how temperature changes impact axial drift within the optical system, providing valuable insights for optimal design strategies in optics. Yin Jing et al. calculated the defocus amount of the infrared zoom lens within the temperature range of −40 °C to 50 °C using Sigfit [13]. Then, an active thermal difference elimination design was implemented according to the analysis results, and the imaging results were found to be satisfactory. Based on the MATLAB numerical computing environment, Scott Roberts utilized the merit function routine (MFR) to evaluate the requirements and performance of the active optics system of the Thirty Meter Telescope [14]. R. Gao carried out a performance analysis of an all-aluminum off-axis three-mirror system using the optomechanical–thermal coupling analysis method [15]. This analysis involved data processing and coordinate transformation. Specifically, the deformation data of the mirror nodes in the global coordinate system obtained from the finite element analysis were converted into XY polynomial coefficients in the local coordinate system within Zemax.

Most scholars primarily focus on the impact of the rigid-body displacement of the main mirror on image quality. In this study, we conduct a static analysis of the primary mirror assembly to determine the surface shape accuracy of the mirror under gravitational forces and temperature variations. We investigate the impact of both rigid-body displacement and elastic deformation of the mirror on the transfer function of the optical system under various operating conditions, employing an integrated optomechanical analysis method. Furthermore, we achieve a dynamic full-link simulation that encompasses all stages from the initial source input to the final response output within the optical machine framework. This analytical approach offers valuable insights into the structural layout and design of the entire satellite system, enabling the analysis results to be more in line with the actual engineering scenarios. By conducting simulation analysis during the design phase, it is possible to shorten the development cycle and reduce costs while ensuring the imaging quality. This methodology is also applicable to image quality assessments in other optical imaging systems, such as near-eye display devices.

## 2. The Impact of Mirror Movement on Image Motion

In this study, we will perform an analysis of a specific high-resolution optical camera. Its optical system employs an off-axis four-mirror arrangement composed of the primary mirror, secondary mirror, folding mirror, tertiary mirror, and quaternary mirror. The vibration of the mirrors at different degrees of freedom exerts varying effects on the image motion within the optical system. Taking a spherical mirror as an example, the radius of the spherical mirror is denoted as r, while the incident height of the parallel beam is represented as h [16]. We will analyze the effects of the tilt on the optical system.

### 2.1. X-Direction Decentered of Spherical Mirror

**Figure 1:**
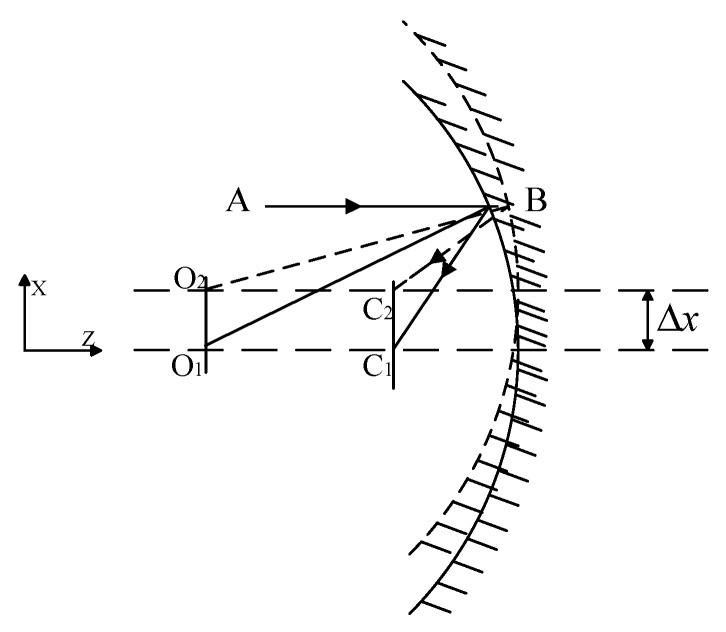
X-direction decentered of spherical mirror.

As illustrated in Figure 1, when the spherical mirror undergoes a translation along the X-direction, referred to as X eccentricity, the geometric imaging relationship indicates that the focal point of the spherical mirror is positioned midway between the sphere’s center and its vertex.

(1) $$\delta_{x}=C_{1}C_{2}=\Delta x$$

Here, *δ_x_* represents the translational movement of the spherical mirror along the X-direction, while Δ denotes the magnitude of image shift induced by this translational movement.

For a general optical telescope, the optical system is symmetric in the OX-direction and the OY-direction, so the amount of image shift caused by the translation along the Y-direction should be the same as that caused by the translation along the X-direction. The analysis shows that the image shift caused by the translation of the mirror in the direction of the vertical optical axis is equal to the amount of mirror translation, and the direction is the same.

### 2.2. Z-Direction Decentered of Spherical Mirror

**Figure 2:**
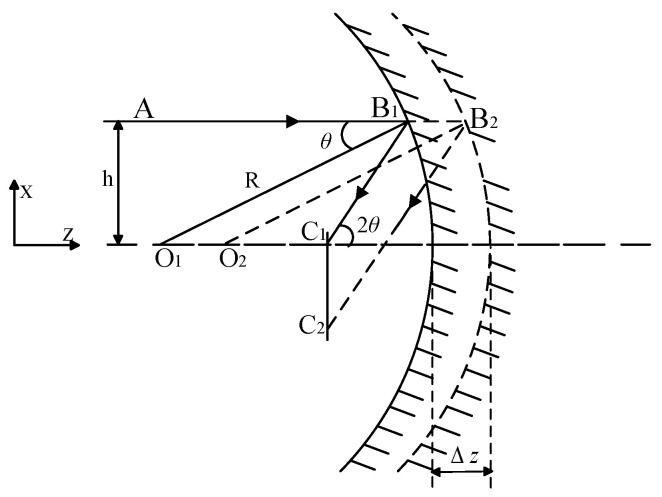
Z-direction decentered of spherical mirror.

When the spherical mirror undergoes translation along the optical axis, i.e., in the Z-direction, both the center of the sphere and the focal point of the spherical mirror shift accordingly, as depicted in Figure 2. The resultant image shift is denoted as δ_z_.

(2) θ≈tanθ≈sinθ=hR

(3) δz=C1C2=Δztan2θ=ΔzR2h

Here, *δ_z_* represents the translational movement of the spherical mirror along the Z-direction, *δ* denotes the magnitude of image shift induced by this translational movement along the Z-direction, and *θ* signifies the angle of incidence.

A linear relationship exists between the translational displacement Δ*z* and the image shift *δz*, where *δz* is influenced by the curvature radius R of the spherical mirror and the incident height *h*.

### 2.3. X-Direction Tilt of Spherical Mirror

**Figure 3:**
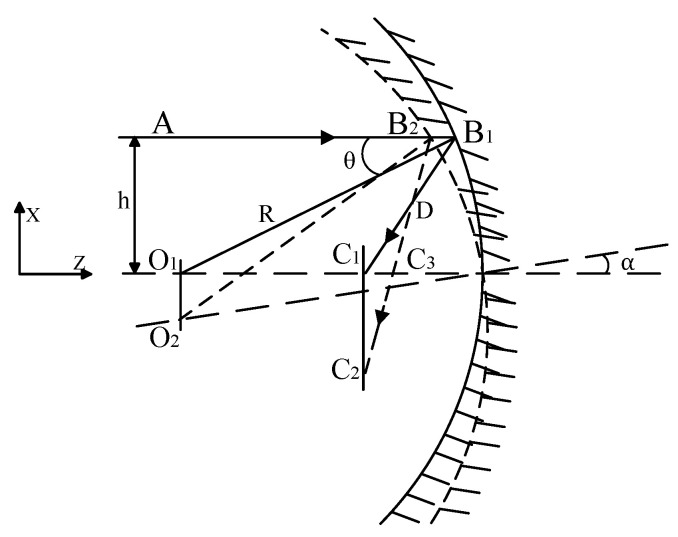
X-direction tilt of spherical mirror.

When the spherical mirror rotates around the *X*-axis, with an angle of tilt denoted as *θ* (as illustrated in Figure 3), the rotation angle of the reflected light is observed to be 2*θ*. The angle induced by the micro-vibrations of the mirror is considered small, allowing us to approximate that the incident points of paraxial light rays B1 and B2 are nearly coincident. So, C1B2≈C1B1=O1C1=R2

(4) $$∠C_{1}C_{3}C_{2}=∠DC_{1}C_{3}+∠C_{1}DC_{3}=2\alpha +2\theta$$

(5) C1C3≈C1D·sin∠C1DC3=R2·2α=Rα

From the analysis presented above, it is evident that the rotation of the spherical mirror around the *X*-axis results in a tilt of the actual image plane relative to the ideal focal plane. This induced image shift is directly correlated with both the radius of the curvature and the angle of the rotation tilt of the spherical mirror.

### 2.4. The Spherical Reflector Is Tilted Around Z

For spherical mirrors, the mirror rotates symmetrically about the *Z*-axis; thus, tilting the mirror around this axis has a minimal impact on the optical system.

Based on this analysis, we can draw several conclusions: The eccentricity of the optical elements along the X- and Y-axis due to micro-vibrations primarily results in a shift in the image point in the same direction on the image plane. Deviations along the *Z*-axis lead to defocus, which increases the size of the dispersion spot for image points. Additionally, oblique vibrations of optical elements around both the X- and Y-axis cause a tilt between the actual image plane and the ideal focal plane. Consequently, what is received by the detector is essentially a projection of an ideal image point, resulting in the movement of that image point across the image plane. Given that optical elements are symmetric with respect to the *Z*-axis, rotation around this axis does not significantly contribute to image degradation.

## 3. Simulation

**Figure 4:**
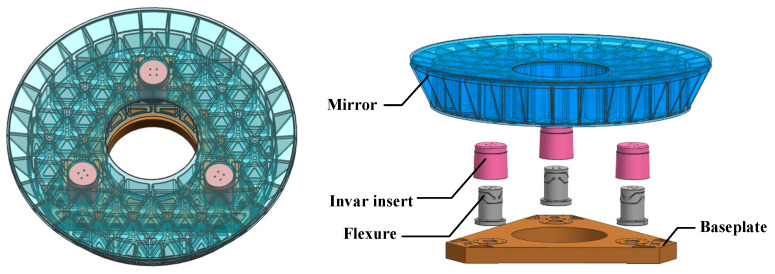
Primary mirror assembly.

The primary mirror of a particular off-axis four-reflector optical telescope has a diameter of 800 mm, as illustrated in Figure 4. In the domain of space optical systems, the selection of mirror materials is governed by several key principles: high specific stiffness and strength, commendable thermal stability, resilience to space environmental conditions, as well as favorable mechanical and optical processability. Considering various factors such as material supply cycles, cost implications, and the aforementioned principles in a comprehensive manner, it has been determined that the gel-casting reaction-sintered RB-SiC—independently developed by the Changchun Institute of Optics, Fine Mechanics and Physics at the Chinese Academy of Sciences—will serve as the primary mirror material. The forming process associated with this SiC material facilitates the fabrication of large-scale mirror bodies with complex geometries. It allows for direct one-step formation of a semi-closed structure on the back surface, thereby significantly enhancing the specific stiffness of the primary mirror. To ensure proper installation and positioning of the SiC primary mirror, it is essential for its body to provide mechanical interfaces and support structures suitable for threaded connections and pin positioning. Typically, metal inserts exhibiting superior mechanical connection properties are bonded into designated installation holes located on the rear side of the primary mirror.

In addressing compatibility concerns regarding linear expansion coefficients between these inserts and the SiC mirror body, low-expansion alloy 4J32 has been selected for use in fabricating these inserts. This alloy possesses an adjustable linear expansion coefficient within a specified temperature range to ensure thermal compatibility with SiC materials. The flexure components connected to these inserts are constructed from an iron alloy (TC4), which is characterized by low density, high specific stiffness, excellent stability under varying conditions, and good machinability. The baseplate connected to the flexure is fabricated from a high-volume fraction SiC particle-reinforced aluminum matrix composite material (SiC/Al). This material features a higher specific stiffness, a low coefficient of linear expansion, high thermal conductivity, and is convenient for processing.

The structural design of the primary mirror encompasses aspects such as the lightweighting pattern of the mirror body, the back closure configuration, the diameter-to-thickness ratio, and the number and location of support holes. Generally speaking, for positioning purposes, circular mirrors do not employ quadrilateral lightweight holes, as these result in an extremely low lightweighting ratio. Under the condition of an equal lightweighting ratio, the stiffness of hexagonal lightweight holes is inferior to that of triangular ones. Therefore, triangular lightweight holes are selected. Regarding the back closure configuration, the fully closed structure exhibits the highest stiffness, followed by the semi-closed structure, and the open structure has the lowest stiffness. Taking into account both manufacturability and the stiffness of the mirror body, a semi-closed structure is adopted.

The diameter-to-thickness ratio of the mirror body is directly correlated with the number and location of support points. The greater the number of support points, the larger the diameter-to-thickness ratio can be, and thus the lower the surface density of the mirror. However, an excessive number of support points can readily lead to over-constraint, causing astigmatic deformation of the mirror. Based on the principle of kinematic constraint, a three-point support method is applied to the mirror. At each support point, a support element with orthogonal two-way flexibility is arranged. This arrangement can not only constrain the six degrees of freedom of the primary mirror to achieve complete positioning but also prevent over-constraint. Additionally, the presence of the flexible element serves to relieve thermal stress. Ultimately, it is decided that the mirror body will utilize triangular lightweight holes, a semi-closed back structure, and a three-point support configuration. The flexure adopts the Cartwheel type. The Cartwheel type flexible hinge, which is the flexible element of the flexible support, offers advantages such as small axial drift, low stress concentration, high anti-buckling capacity, and ease of processing. The baseplate linked to these flexure components is manufactured from a high-volume fraction SiC particle-reinforced aluminum matrix composite material. The flexure is connected to the mirror via an invar insert and secured to the baseplate using screws.

As a key component of the optical telescope, it not only requires outstanding dynamic characteristics but must also guarantee extremely high surface shape accuracy under various external conditions, namely good static characteristics. As the optical element with the largest diameter in the entire optical system, any shape error on the surface of the primary mirror will directly influence the imaging quality of the system.

**Figure 5:**
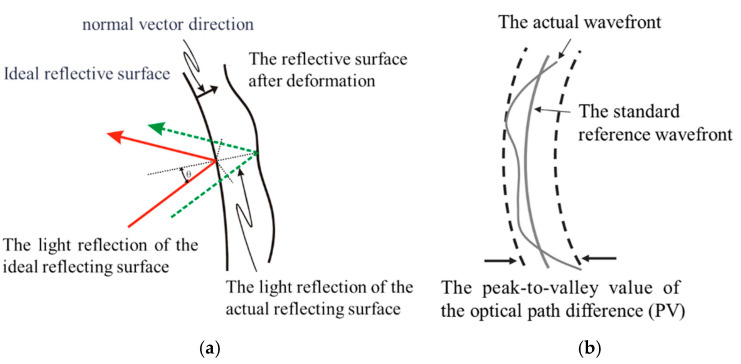
The actual reflective surface with errors and the light wavefront with errors after reflection: (**a**) the influence of the error of the reflective surface on the light reflection; (**b**) the actual light wavefront with errors.

Due to light’s wave–particle duality, light emitted from a point source (ideal spherical wave) is reflected by the mirror; however, this reflection results in a wavefront difference caused by surface shape errors on the reflecting mirror, as illustrated in Figure 5 [17].

A static analysis was conducted on the large-diameter primary mirror. It is essential to establish a finite element model of the primary reflector. The optical performance of the primary reflector was analyzed separately under different gravitational conditions in the X/Y/Z-directions and a temperature increase of 5 °C. To meet the imaging quality and structural strength requirements, the accuracy of the primary mirror’s surface shape must satisfy RMS < 15 nm (λ/40) and PV < 63 nm (λ/10) when subjected to its own weight in three orthogonal directions and a steady-state temperature rise of 5 °C, where λ = 632.8 nm.

**Figure 6:**
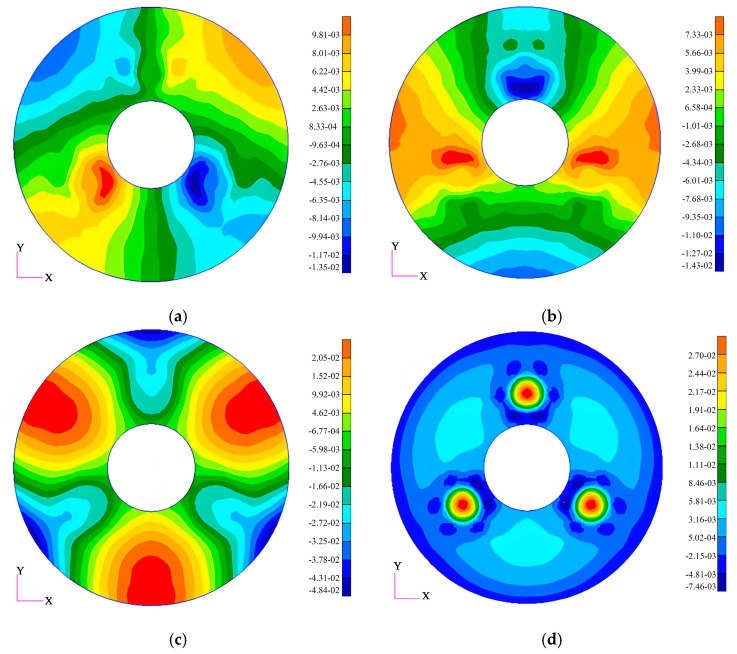
The primary mirror nephogram under different conditions: (**a**) X-direction 1 G gravity deformation; (**b**) Y-direction 1 G gravity deformation; (**c**) Z-direction 1 G gravity deformation; (**d**) 5 °C temperature rise without gravity.

In the subsequent section of this study, we will perform relevant static analyses on the primary mirror. The analysis results indicate that under the conditions of applying 1 G gravitational force in the X-, Y-, and Z-directions and a temperature increase of 5 °C (in the absence of gravitational effects), the surface form accuracies of the mirror are 3.37 nm, 3.07 nm, and 12.05 nm, respectively. Figure 6 depicts the surface form contour plots of the mirror under each of the above working conditions (after the removal of rigid-body displacements).

In view of the fact that we intend for the subsequent optical–mechanical integration analysis to comprehensively assess the joint influence of the rigid-body displacement of the mirror and the mirror deformation on the imaging quality of the optical system, Table 1 compiles the rigid-body displacement magnitudes and surface deformation data of the mirror in three orthogonal directions under various working conditions. Herein, the rigid-body displacement encompasses translational motions along the three coordinate axes (eccentricity in the X-direction, eccentricity in the Y-direction, offset in the Z-direction) and rotational motions about the three axes (tilt in the X-direction, tilt in the Y-direction, tilt in the Z-direction).

**Figure 7:**
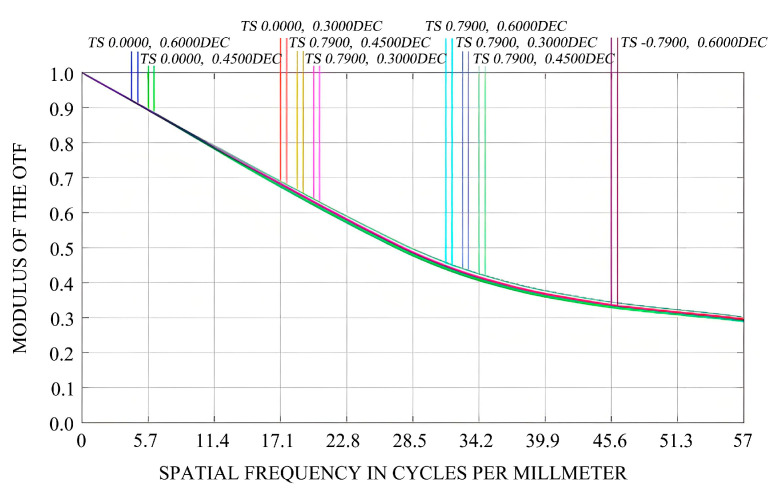
Initial design of the MTF.

**Figure 8:**
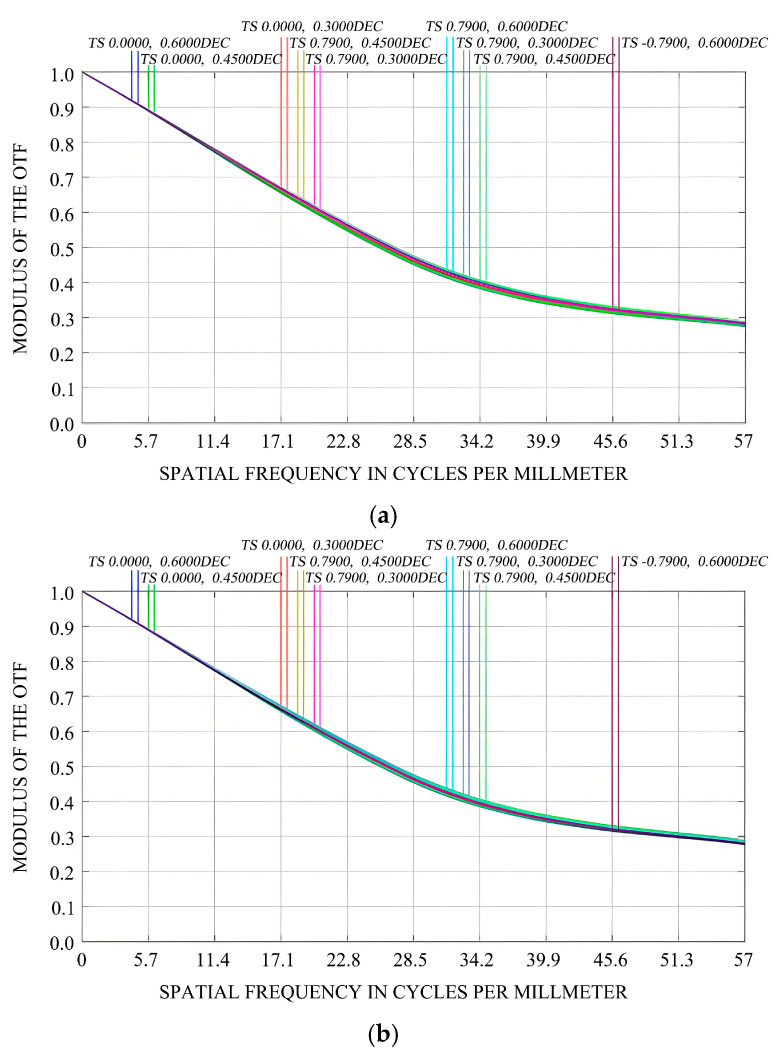

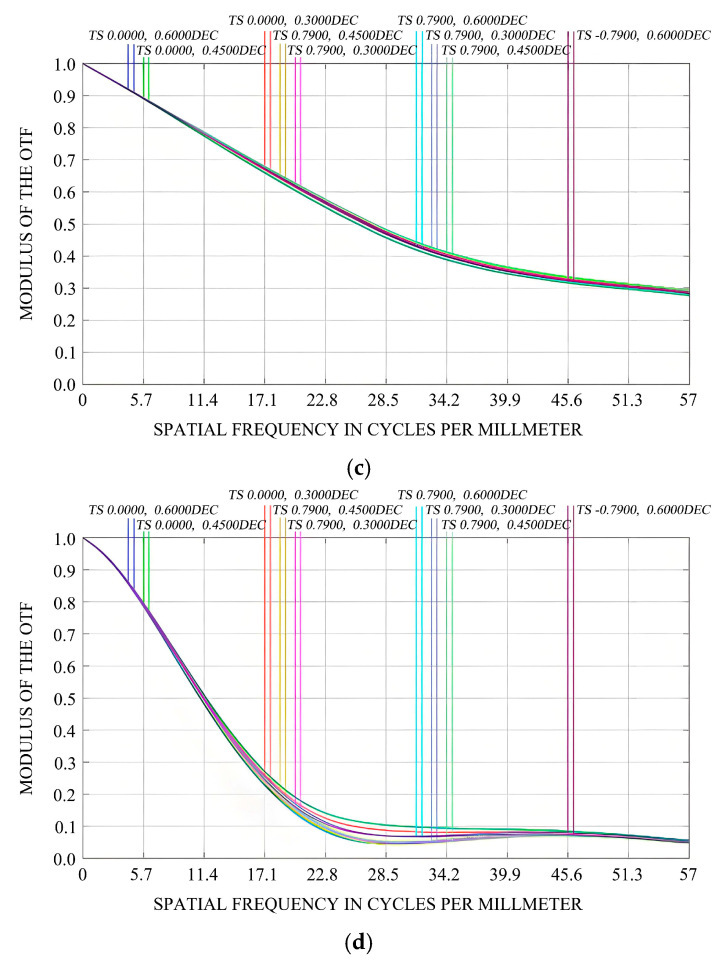
Influence of different working conditions on the MTF. (**a**) The impact of 1 G gravity in the X-direction on the MTF; (**b**) The impact of 1 G gravity in the Y-direction on the MTF; (**c**) The impact of 1 G gravity in the Z-direction on the MTF; (**d**) Influence of 5 °C temperature rise (without gravity) on the MTF.

This study considers not only the impact of mirror surface deformation on the imaging quality of optical systems but also performs a collaborative analysis of both rigid-body displacement and mirror surface deformation of the mirror assembly. The primary mirror’s surface is fitted using Sigfit, and the resulting files (.zpl files) containing data on rigid-body displacement and mirror surface deformation from this analysis are imported into ZEMAX. Within ZEMAX, we visually analyze how the rigid-body displacement and mirror surface deformation of the primary mirror affect the modulation transfer function (MTF) under 1 G gravity in the X/Y/Z-directions, as well as under a temperature increase of 5 °C, as illustrated in Figure 7 and Figure 8.

The modulation transfer function (MTF) curve is intended to assess the imaging quality of an optical system and serves as a crucial metric for evaluating such quality. The ordinate represents the transfer function, which can be interpreted as contrast, while the abscissa represents line pairs per millimeter (lp/mm), which can be regarded as resolution. Here, ‘S’ denotes the sagittal plane, and ‘T’ represents the meridian plane. Specifically, the MTF quantifies the degree of attenuation of the contrast (amplitude) of sinusoidal intensity distribution functions at various frequencies after being imaged by an optical system. When the contrast of a particular frequency reaches zero, it implies that there is no change in the light intensity of that frequency, indicating that the frequency has been cut off. The abscissa represents frequency, and the ordinate represents the normalized contrast.

Figure 7 depicts the initial MTF of the optical imaging system. From this figure, it can be observed that the MTF curve is smooth, and the discrepancy between the sagittal and meridional curves across different fields of view is minimal, suggesting the presence of minor aberrations. At the Nyquist frequency, the MTF value is approximately 0.3, fulfilling the design specifications. Figure 8a–c illustrate the variations in the MTF values of the optical imaging system under the influence of 1 G gravitational force in the X-, Y-, and Z-directions, respectively. Evidently, under 1 G gravitational force in these three directions, the rigid-body displacements and surface shape errors of the mirror have negligible impacts on the MTF of the optical system. The MTF values at the Nyquist frequency still satisfy the design requirements. Figure 8d demonstrates that under the working condition of a 5 °C temperature increase (in the absence of gravitational force), the rigid-body displacements and surface shape errors of the mirror have a substantial influence on the MTF of the optical system. The difference between the sagittal and meridional curves in different fields of view becomes more pronounced, indicating significant aberrations. Table 2 presents the changes in the MTF values of the sagittal and meridional planes in different fields of view. Taking the (0°, 0.6°) field of view and the meridional plane as an example, at the Nyquist frequency, the MTF deteriorates from the design value of 0.301060 to 0.056873.

The analysis results show that due to the significant impact of temperature changes on surface accuracy, temperature variations can lead to the degradation of the MTF. Therefore, more precise temperature control can be adopted to mitigate the influence of temperature changes and further ensure imaging quality.

## 4. Optical Detection Experiment of the Primary Mirror

To assess the impact of gravity on the surface shape accuracy of the primary mirror assembly, it is essential to perform a gravity variation test on a mirror exhibiting high surface shape precision. Optical satellites are calibrated and evaluated within the Earth’s gravitational field, where all structural components experience a vertical gravitational force of 1 G. The alteration in the orientation of the primary mirror itself within this gravitational field can effectively substitute for changes in the direction of the gravitational field relative to the primary mirror. In a detection state with a horizontal optical axis, this change is accomplished by rotating the primary mirror around its own optical axis by a specified angle.

The optical path for detection was set up within a constant-temperature assembly and adjustment chamber. The ambient temperature was maintained at (22 ± 1) °C. The optical path base is a steel air-floating vibration isolation platform developed by the China Electronics Engineering Design and Research Institute. The interferometer employed is the DynaFiz model manufactured by ZYGO Corporation in the Connecticut of United States. The primary mirror assembly was installed in the main load-bearing structure of the remote sensor in its emission state, and the main load-bearing structure was mounted on a tooling fixture. To measure the surface topography of the aspheric primary mirror, a null compensation testing method was utilized. A null compensator was placed between the interferometer and the mirror surface in order for aspheric wavefront inspections to be performed. Figure 8 presents the on-site optical inspection of the aspheric primary mirror and the surface topography map of the inspection results.

**Figure 9:**
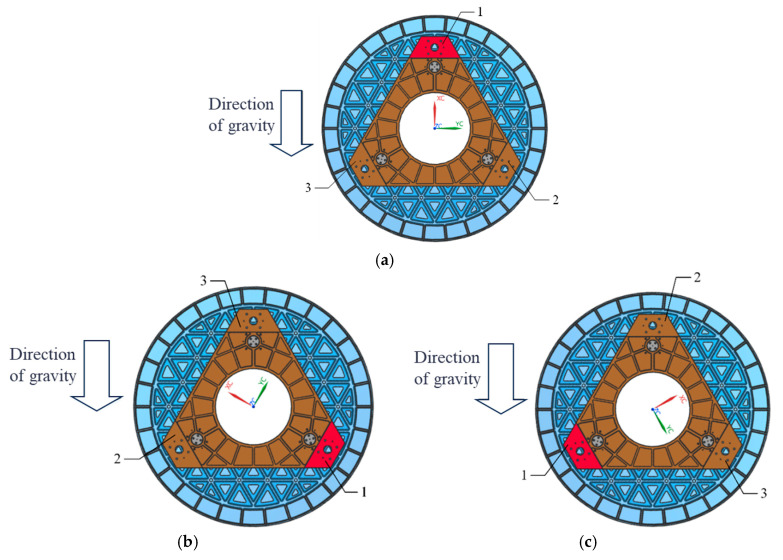
The test mirror is rotated around the optical axis when the optical axis is horizontal: (**a**) 0°; (**b**) 120°; (**c**) 240°.

**Figure 10:**
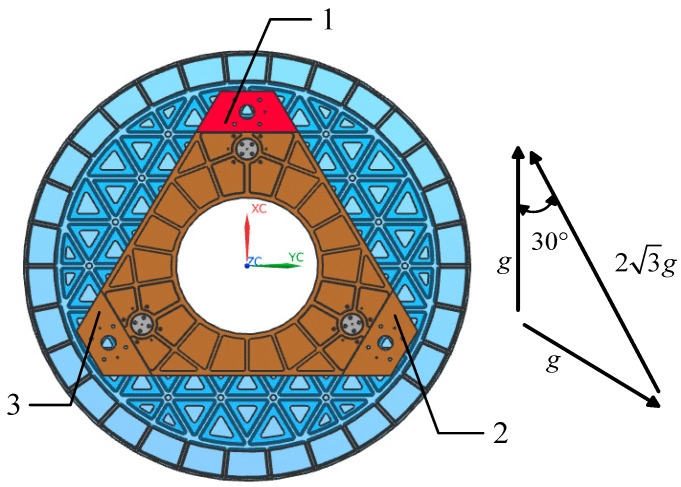
The equivalent gravity field change after the test mirror is rotated 120°.

Initially, the mirror surface is processed according to the direction illustrated in Figure 9a (designated as 0°) until a surface shape accuracy of λ/40 (where λ = 632.8 nm) is achieved. Subsequently, the mirror is rotated 120° about its optical axis, as depicted in Figure 9b, while Figure 9c illustrates its position after being rotated an additional 240° around that same axis. As rotation occurs around its optical axis, there are corresponding changes in its force state. Taking a rotation of 120° as an example, Figure 10 presents its force state post-rotation. Conducting an optical detection of the surface shape before and after this change in the force state will provide insights into the variations in the accuracy of the surface shape experienced by the primary mirror under radial gravitational forces.

**Figure 11:**
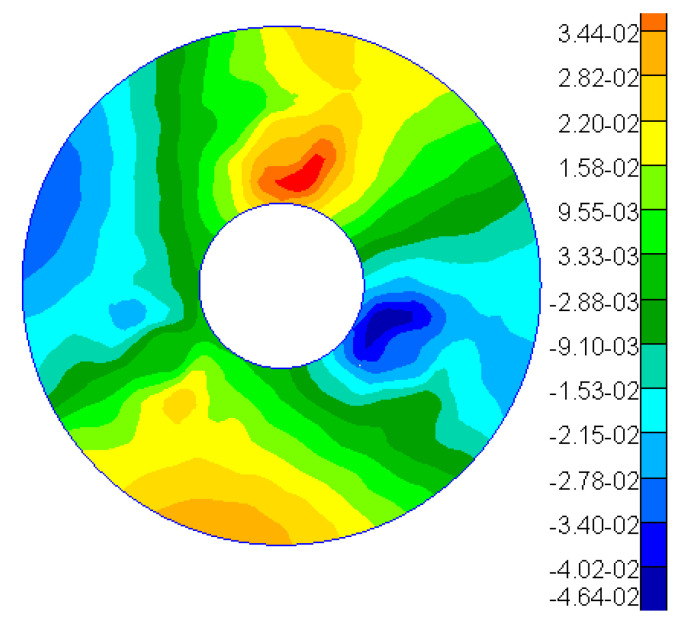
Mirror surface shape analysis results.

Based on the aforementioned theory, an optical–mechanical integration analysis of the primary mirror assembly was conducted. The applied load is illustrated in Figure 10, and the simulation results indicate that the surface accuracy of the mirror under this gravitational field measures 11.69 nm. Figure 11 presents a contour map depicting the Zernike polynomial fitting of the nodal displacements of the mirror surface, with rigid-body displacements (bias and tilt) and the power term subtracted. Table 3 lists the first seven coefficients derived from the Zernike polynomial fitting of the mirror surface displacement results. The analysis reveals that under this gravitational field, the astigmatism term is notably significant.

**Figure 12:**
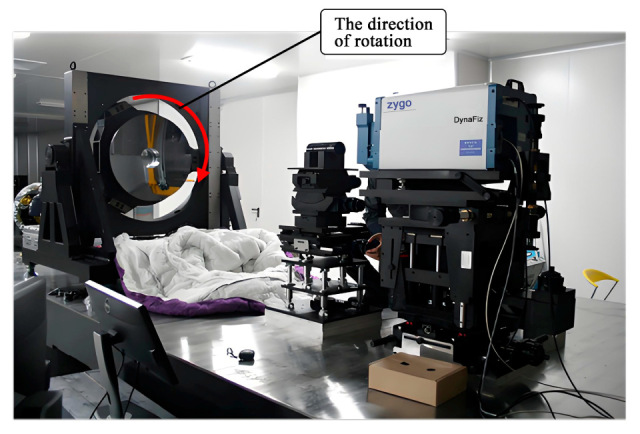
Optical setup.

**Figure 13:**
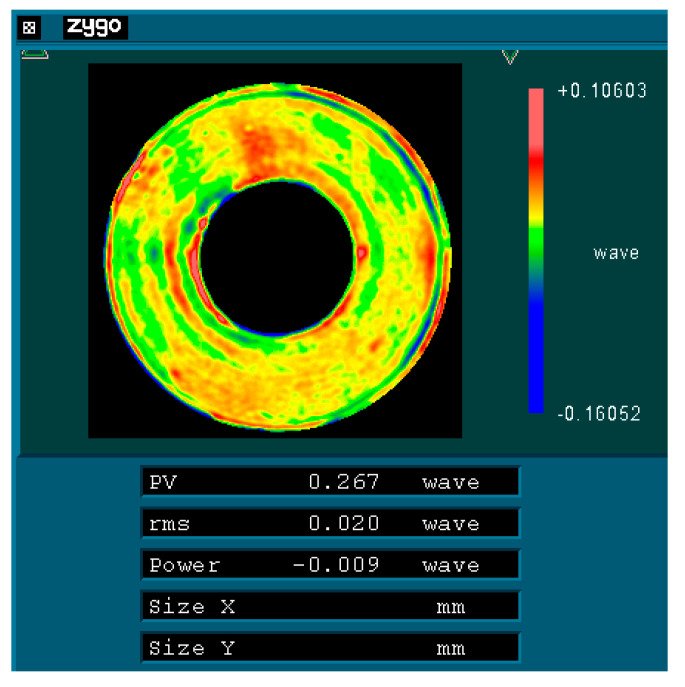
Surface shape detection results of the test mirror in the 0° direction.

**Figure 14:**
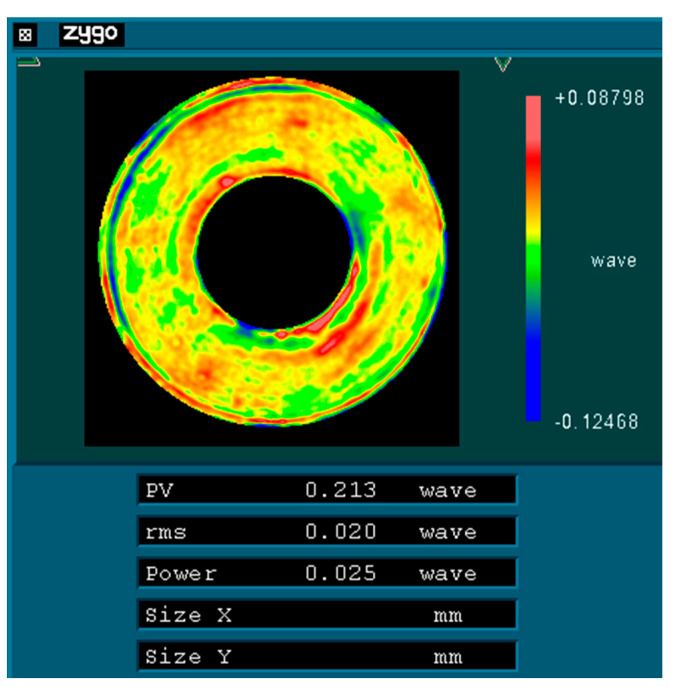
Surface shape detection results of the test mirror in the 120° direction.

**Figure 15:**
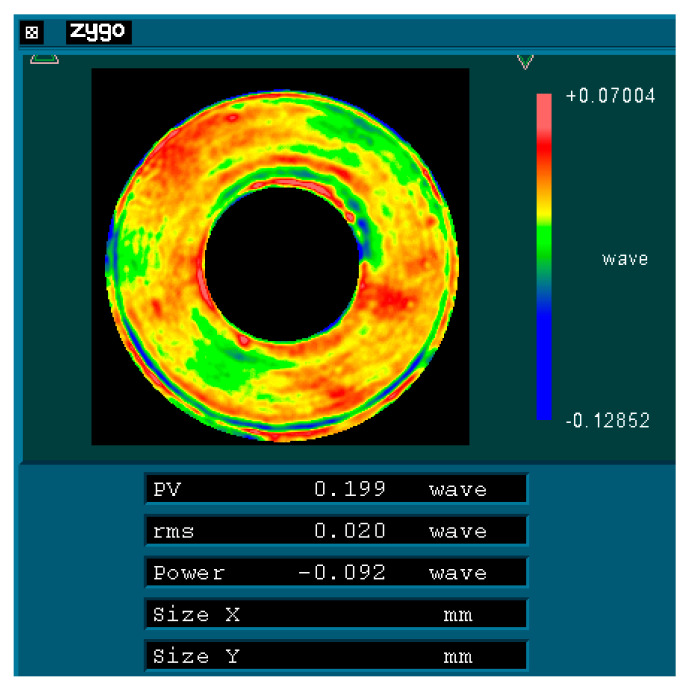
Surface shape detection results of the test mirror in the 240° direction.

To test the primary mirror with interferometry, the typical aspheric refractive Offner zero-position compensation assembly has been adopted [18]. The zero-position compensator for the detection optical path was designed and fabricated based on the parameters of the aspheric mirror. The helium–neon (He-Ne) laser plane wave emitted by the phase-shifting interferometer passes through the Offner compensator and is transformed into an aspheric wavefront matching the aspheric surface of the test mirror under examination. After being reflected by the aspheric surface under detection, it returns to the interior of the interferometer and interferes with the reference wavefront generated by the beam splitting to produce fringes. Figure 12 represents the optical detection test on-site, and Figure 13, Figure 14 and Figure 15 show the optical detection results.

As illustrated in Figure 10 and Figure 11, after the mirror is rotated by 120°/240°, its RMS value remains at 0.02 λ (where λ = 632.8 nm), equivalent to 12.66 nm. This observation indicates that the main mirror assembly exhibits good static performance. The simulation results indicate that the RMS value of the mirror following a rotation of 120° is measured at 11.69 nm, resulting in a relative error of 7.66% when compared to the experimental findings. Given that the main mirror demonstrates robust static performance, the surface shape error induced by gravitational effects is minimal, thereby exerting a negligible influence on the MTF (modulation transfer function). This further corroborates the accuracy of our simulation analysis.

## 5. Conclusions

We performed a static analysis of the main mirror assembly, yielding the surface shape accuracy of the mirror under gravitational forces and temperature variations. Furthermore, an integrated optical and mechanical analysis method was employed to examine the impact of rigid-body displacement and elastic deformation of the mirror on the transfer function of the optical system across various operating conditions. The results of our analysis indicate that under gravitational influences in the X/Y/Z-directions, the mirror retains commendable optical performance, with minimal rigid-body displacement and surface shape errors that exert little effect on the modulation transfer function (MTF) of the optical system. Conversely, when subjected to fluctuating temperatures, significant rigid-body movement and deformation occur within the mirror itself, leading to MTF degradation for knife-edge optical systems. For instance, in a field of view defined by (0 degrees, 0.6 degrees) along with its meridian plane at Nyquist frequency, the MTF deteriorates from its design value of 0.301060 to 0.056873. Optical detection tests have been performed on the main mirror assembly; the results demonstrate that it maintains favorable static properties even under gravitational influence. Consequently, this ensures that the imaging quality remains intact despite changes in gravitational fields, further validating our simulation analyses’ accuracy. This analytical approach can offer valuable insights into the structural layout and design of the entire satellite. Conducting a simulation analysis during the design phase not only reduces costs and shortens the development cycle but also ensures that the imaging is of a high quality.

## Figures and Tables

**Table 1:** The rigid-body displacement and mirror surface shape error of the mirror under gravity and temperature rise.

Working Condition		X-Direction Gravity	Y-Direction Gravity	Z-Direction Gravity	5° Temperature Rise
Rigid-body displacement	X-direction decenter/mm	3.0954 × 10^−3^	−4.3830 × 10^−6^	−1.2584 × 10^−8^	−3.6754 × 10^−7^
	Y-direction decenter/mm	4.3635 × 10^−6^	3.0954 × 10^−3^	2.4336 × 10^−9^	1.0504 × 10^−8^
	Z-direction offset/mm	−7.0699 × 10^−9^	2.2158 × 10^−9^	2.4959 × 10^−3^	−6.9990 × 10^−3^
	X-direction tilt/rad	1.4354 × 10^−7^	−5.7964 × 10^−7^	2.8166 × 10^−11^	4.5731 × 10^−12^
	Y-direction tilt/rad	5.7969 × 10^−7^	1.4363 × 10^−7^	1.2095 × 10^−10^	−1.4364 × 10^−9^
	Z-direction tilt/rad	−6.9126 × 10^−11^	−1.4330 × 10^−10^	−1.5188 × 10^−7^	1.1528 × 10^−8^
RMS/mm		3.374 × 10^−6^	3.374 × 10^−6^	3.067 × 10^−6^	1.252 × 10^−5^

**Table 2:** The influence of 5 °C temperature rise (without gravity) on the MTF.

Nyquist Frequency	View Field	Initial Design MTF		5 °C Temperature Rise (Without Gravity)	
		Sagittal Plane	Meridian Plane	Sagittal Plane	Meridian Plane
57 lp/mm	0.0000, 0.6000	0.296985	0.301060	0.055947	0.056873
	0.0000, 0.4500	0.292894	0.302631	0.054647	0.056542
	0.0000, 0.3000	0.298536	0.300621	0.052097	0.056273
	0.7900, 0.4500	0.302067	0.302077	0.057842	0.056861
	0.7900, 0.3000	0.301605	0.301747	0.055068	0.055523
	0.7900, 0.6000	0.292625	0.293102	0.049947	0.049892
	−0.7900, 0.3000	0.301605	0.301747	0.055947	0.056245
	−0.7900, 0.4500	0.302067	0.302077	0.055947	0.055573
	−0.7900, 0.6000	0.292625	0.293102	0.049985	0.049625

**Table 3:** Zernike coefficient of mirror deformation fitting of the main reflector under the action of gravity.

Aberration Types	Amplitude/λ	Phase/°
Shift	0.00000	0.0
Tilt	0.00085	119.9
Defocus	0.00000	0.0
First-order astigmatism	0.03732	74.4
First-order coma	0.01585	−60.2
First-order spherical aberration	0.00000	46.5
First-order trefoil	0.00000	0.0

## Data Availability

Data underlying the results presented in this study are not publicly available at the time of publication, but they may be obtained from the authors upon reasonable request.
